# AGE DIFFERENCES IN THE CORRELATES OF PAIN AND QUALITY OF LIFE AMONG BREAST AND COLORECTAL CANCER SURVIVORS

**DOI:** 10.1093/geroni/igae098.0994

**Published:** 2024-12-31

**Authors:** Jessica Krok-Schoen, Melica Nikahd, Madison Hyer, Elizabeth Arthur, Erin Stevens, Theodore Brasky, Leorey Saligan, Diane Von Ah

**Affiliations:** The Ohio State University, Columbus, Ohio, United States; The Ohio State University, Columbus, Ohio, United States; The Ohio State University, Columbus, Ohio, United States; The Ohio State University Comprehensive Cancer Center, Columbus, Ohio, United States; The Ohio State University Comprehensive Cancer Center, Columbus, Ohio, United States; The Ohio State University College of Medicine, Columbus, Ohio, United States; Symptoms Biology Unit, Division of Intramural Research, Bethesda, Maryland, United States; The Ohio State University, Columbus, Ohio, United States

## Abstract

Few studies evaluated the differential impact of cancer-related symptoms in older patients compared to younger patients. This national cross-sectional study explored the age differences (21-64 vs. ≥65 years) in the demographic, health, and social correlates of pain and quality of life (QoL) among 621 breast and colorectal cancer survivors. Median pain and QoL scores did not differ between the younger and older groups (p-values = 0.80, 0.16, respectively). Higher pain for the whole sample was associated with more education (β = 5.3, p = 0.02) and higher perceived stress (β = 2.4, p < 0.001). Higher QoL was associated with employment (β = 0.8, p = 0.003), more social support (β = 0.4, p < 0.001, 5-point increase), and advanced cancer stage (β = 0.7, p = 0.04). The association of QoL and marital status differed by age; younger, married participants reported higher QoL, and older, married participants had lower QoL (p = 0.003). Among younger and older cancer survivors, our results indicate more similarities than differences in the symptom burden.

